# Distribution of NECAB1-Positive Neurons in Normal and Epileptic Brain—Expression Changes in Temporal Lobe Epilepsy and Modulation by Levetiracetam and Brivaracetam

**DOI:** 10.3390/ijms26104906

**Published:** 2025-05-20

**Authors:** Krisztina Kelemen, Károly Orbán-Kis, Ádám Szentes, Zsolt András Nagy, Hanga Kelemen, Anna Fehér, László-István Bába, Zsolt Gáll, Eszter Horváth, István Katona, Szabolcs Szatmári, József Attila Szász, Tibor Szilágyi

**Affiliations:** 1 Department of Physiology, George Emil Palade University of Medicine, Pharmacy, Science, and Technology of Targu Mures, 540142 Târgu Mureș, Romania; krisztina.kelemen@umfst.ro (K.K.); tibor.szilagyi@umfst.ro (T.S.); 2 Doctoral School, George Emil Palade University of Medicine, Pharmacy, Science, and Technology of Targu Mures, 540142 Târgu Mureș, Romania; 3 Molecular Neurobiology Research Group, HUN-REN Institute of Experimental Medicine, Szigony st 43, H-1083 Budapest, Hungary; 4 Faculty of Medicine, George Emil Palade University of Medicine, Pharmacy, Science, and Technology of Targu Mures, 540142 Târgu Mureș, Romania; 5 Translational Behavioural Neuroscience Research Group, HUN-REN Institute of Experimental Medicine, Szigony st 43, H-1083 Budapest, Hungary; 6 János Szentágothai Neurosciences Division, Doctoral College, Semmelweis University, Ulloi st 26, H-1085 Budapest, Hungary; 7 Department of Pharmacology and Clinical Pharmacy, George Emil Palade University of Medicine, Pharmacy, Science and Technology of Targu Mures, 540142 Târgu Mures, Romania; 8 Department of Psychological and Brain Sciences, Indiana University Bloomington, 702 N Walnut Grove Ave, Bloomington, IN 47405-2204, USA; 9 Department of Neurology, George Emil Palade University of Medicine, Pharmacy, Science, and Technology of Targu Mures, 540142 Târgu Mureș, Romania

**Keywords:** calcium-binding proteins, NECAB1, temporal lobe epilepsy, amygdala, paraventricular nucleus of the thalamus, endopiriform nucleus, hippocampus, SV2A associated antiseizure treatment, levetiracetam, brivaracetam

## Abstract

Calcium-binding proteins (CaBPs) are known to modulate neuronal excitability and calcium signaling, and they may play a role in the imbalances of excitation and inhibition of temporal lobe epilepsy (TLE). While parvalbumin and calretinin are well-characterized CaBPs, N-Terminal EF-Hand Calcium-Binding Protein 1 (NECAB1) remains understudied in epilepsy, despite its association with neurodegenerative conditions. In this study, we used fluorescent immunolabeling to determine the distribution of NECAB1, as well as its co-expression with parvalbumin and calretinin, in brain regions associated with the epileptic circuitry using a kainic acid-induced TLE model. Additionally, we examined the impact of levetiracetam and brivaracetam on NECAB1 expression. In our study, NECAB1-positive cells were prominently localized to the paraventricular nucleus of the thalamus (PVT), endopiriform nucleus (EPN), and amygdala in healthy brain regions involved in epileptic circuitry. A NECAB1–calretinin co-expressing subpopulation was detected in the amygdala, PVT, and hippocampus but was nearly absent in the EPN. In chronic epilepsy, NECAB1 expression was significantly upregulated in the PVT and bilaterally in the amygdala. These findings suggest that NECAB1 upregulation may compensate for epileptic hyperexcitability, potentially contributing to circuit remodeling via thalamocortical regulation and interneuron diversity. Levetiracetam and brivaracetam treatments partially reduced the NECAB1 density increase in TLE, indicating a modulatory effect on NECAB1 expression.

## 1. Introduction

The balance between excitation and inhibition in neural circuits is a fundamental aspect of brain function, and its disruption has long been implicated in the pathophysiology of epilepsy [1,2].

Due to the complexity and diversity of epilepsy, identifying all underlying mechanisms remains challenging. A comprehensive understanding of the syndrome requires a multi-level approach, dissecting its components at the cellular and molecular levels, and subsequently integrating these findings into a broader context of circuit-level changes.

Temporal lobe epilepsy (TLE) is the most prevalent form of focal epilepsy, frequently resistant to pharmacological treatment [3,4]. Consequently, significant efforts have been directed toward systematically deciphering the molecular and cellular mechanisms underlying its pathogenesis [5]. Inhibitory interneurons play a crucial role in maintaining excitatory–inhibitory balance in the healthy brain [6]. During epileptogenesis, structural and functional alterations also impact interneurons; however, the specific contribution of different interneuronal subtypes to pathological changes remains incompletely characterized [7].

Several proteins serve as reliable markers for distinct interneuron types. Among these, calcium-binding proteins (CaBPs) have emerged as valuable markers for identifying and characterizing interneuron populations [8]. However, the role of CaBPs extends beyond their long-standing use as interneuron markers as they are key regulators of intracellular calcium homeostasis and signaling pathways [9]. Given the necessity for precise calcium signaling regulation, CaBPs show molecular and anatomical diversity, as well as varying Ca^2+^-binding kinetics, which suggest their involvement in cell-type-specific modulation of neuronal excitability and synaptic transmission [10], essential for the computational functions of neural circuits [11]. These functions are known to be substantially impaired by TLE; however, they are probably not only consequences but also drivers of epileptogenesis, making them potential therapeutic targets.

Despite the extensive characterization of classical CaBPs such as parvalbumin (PV) and calretinin (CR), the expression and functional roles of many other CaBPs, including NECAB1 (N-Terminal EF-Hand Calcium-Binding Protein 1), remain poorly understood. Consequently, their presence, relative proportions, and functional significance have not yet been characterized. There is an urgent need to investigate the spatial distribution of individual cells that co-express distinct combinations of key calcium-binding proteins. In silico data suggest that such cells are rare and, as a result, have remained largely understudied [12]. A detailed mapping of these neurons, with a particular focus on their colocalization patterns, is therefore essential to better understand their role in neural circuits. Changes in NECAB1 have previously been linked to several pathologies like Alzheimer’s disease, Parkinson’s disease, and related synucleinopathies as well as developmental language disorders [13,14,15]. Furthermore, NECAB1 has been listed among potential epilepsy-associated genes [16]. As the potential redistribution of several CaBPs throughout the pathological circuitry in response to epilepsy is yet to be determined [17], we try to address the underexplored role of NECAB1 in TLE, hypothesizing that its expression and distribution patterns may reveal novel insights into epileptic circuitry.

A critical aspect of epilepsy research is determining how novel antiseizure medications (ASMs) impact the distribution of neuronal populations throughout brain regions, potentially modulating epileptogenesis and altering disease progression [18]. Understanding how ASMs affect NECAB1 and its co-expressed markers could inform both mechanistic and therapeutic perspectives in TLE. Importantly, SV2A-binding ASMs such as levetiracetam and brivaracetam have shown not only anticonvulsant properties but also potential disease-modifying effects, possibly through their role in preserving or restoring synaptic integrity [19,20]. These drugs may indirectly modulate synaptic activity to reduce excitotoxicity and mitigate seizure-induced neuronal injury by influencing the calcium-buffering capacity of CaBPs, including NECAB1.

In this study, we aimed to characterize the expression and distribution of NECAB1 in both healthy and epileptic brains using the intracerebroventricularly injected kainic acid (KA) model of TLE. Through immunohistochemical analysis, we systematically mapped NECAB1-positive cells in brain regions associated with the epileptic circuitry [21], providing novel insights into the neuronal changes that accompany the disease. Additionally, we examined the co-expression between NECAB1 and the well-characterized CaBPs, PV and CR, to better understand their interrelated role within these neural networks. Furthermore, we explored the effects of levetiracetam and brivaracetam on the expression of these CaBPs, offering a new perspective on their potential involvement in therapeutic interventions.

## 2. Results

### 2.1. Mapping of NECAB1 Expression and Its Colocalization with PV and CR in Physiological Conditions Along the Epileptic Circuitry

**Figure 1:**
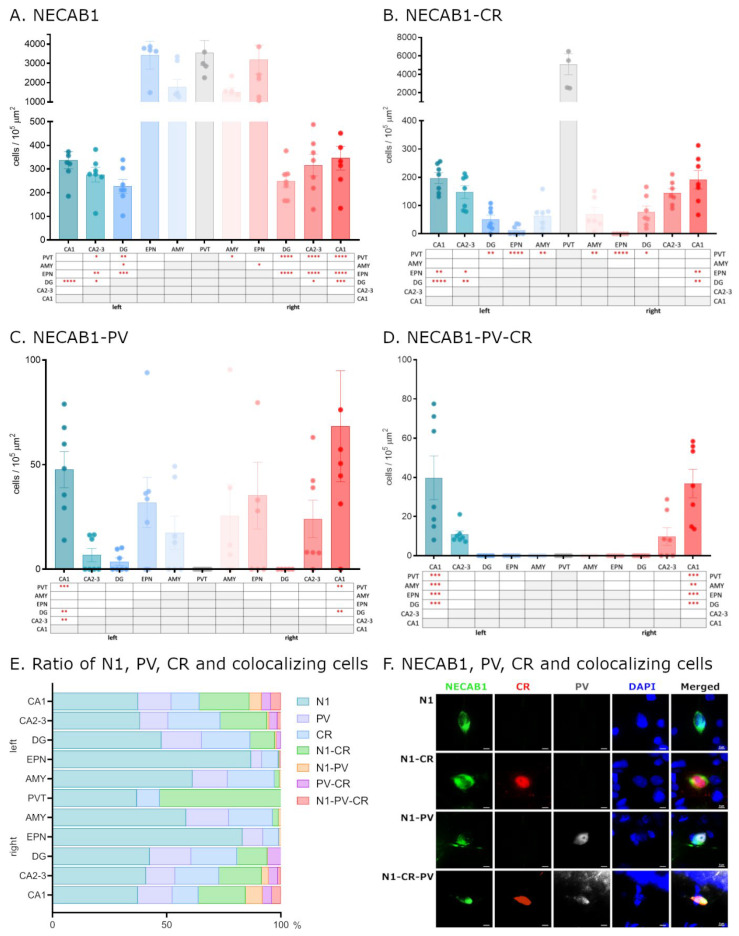
Distribution of NECAB1, PV, and CR cells in the epileptic circuitry. Mapping of cells expressing only NECAB1, as well as NECAB1 and PV and/or CR in physiological conditions, in brain regions known to be involved in the epileptic circuitry (**A**–**D**). The ratio of all CaBP markers’ expressing cells in different brain regions of the epileptic circuitry (**E**). Single channel and composite immunofluorescence colocalization analysis of NECAB1/PV/CR in cells (**F**). N1 = NECAB1-positive cell; N1-PV = NECAB1–PV colocalizing cell; N1-CR = NECAB1–CR colocalizing cell; N1-PV-CR = NECAB1–PV–CR colocalizing cell. An alpha value of 0.05 was used as the cutoff for significance, and we used the following notations: * *p* < 0.05, ** *p* < 0.01, *** *p* < 0.001, and **** *p* < 0.0001.

To elucidate the distribution of calcium-binding proteins, we analyzed the distinct expression patterns of NECAB1, PV, and CR under physiological conditions, focusing on brain regions implicated in epileptic circuitry or possibly affected by seizures. First, we studied the density of neurons expressing each protein individually and compared distributions across brain regions (Figure 1A–E). Neurons exclusively expressing NECAB1 were more abundant in the paraventricular nucleus of the thalamus, endopiriform nucleus, and amygdala (albeit not significantly in the latter) when compared to the hippocampus. Within the hippocampus, significant differences in NECAB1+ cell density were observed among the CA1 and CA2–CA3 subregions in comparison to the dentate gyrus (Figure 1A).

Our findings regarding the density of NECAB1 raise an intriguing question regarding the distribution of individual neurons co-expressing different combinations of major calcium-binding proteins in the healthy brain (Figure 1F).

To determine whether NECAB1+ cells are also PV+ or CR+, we performed colocalization analysis, revealing that a subset of NECAB1-immunopositive neurons also expressed calretinin. These cells were present in all examined brain regions with quantified distributions presented in Table 1, albeit at significantly lower densities compared to the solely NECAB1-expressing cells that did not co-express calretinin.

Neurons co-expressing NECAB1 and calretinin exhibited significantly higher density in the PVT compared to other examined regions. In the hippocampus, a significant difference was observed along the CA1–GD axis, with lower density in the DG relative to CA1 and CA2–CA3 (Figure 1B).

In contrast to NECAB1–CR cells, NECAB1–PV colocalization was observed to a much lesser extent, and only in the CA1 and CA2–3 regions of the hippocampus. NECAB1–PV co-expressing cells were completely absent from the PVT (as were the solely PV-positive cells) but were more abundant in the endopiriform nucleus, amygdala, and hippocampus. Within the hippocampus, their distribution exhibited significant regional differences, displaying a similar decreasing trend along the CA1–GD axis, as in the case of NECAB1–CR cells (Figure 1C).

Only a small number of neurons co-expressing all three CaBPs (NECAB1, PV, and CR) were detected, and these were restricted to the CA1 and CA2–CA3 regions of the hippocampus, with a significantly higher density observed in CA1 (Figure 1D,E).

### 2.2. Epilepsy Increases the Density of NECAB1+ Cells in Several Brain Regions, Which Is Attenuated by the Antiseizure Medications Brivaracetam and Levetiracetam

**Figure 2:**
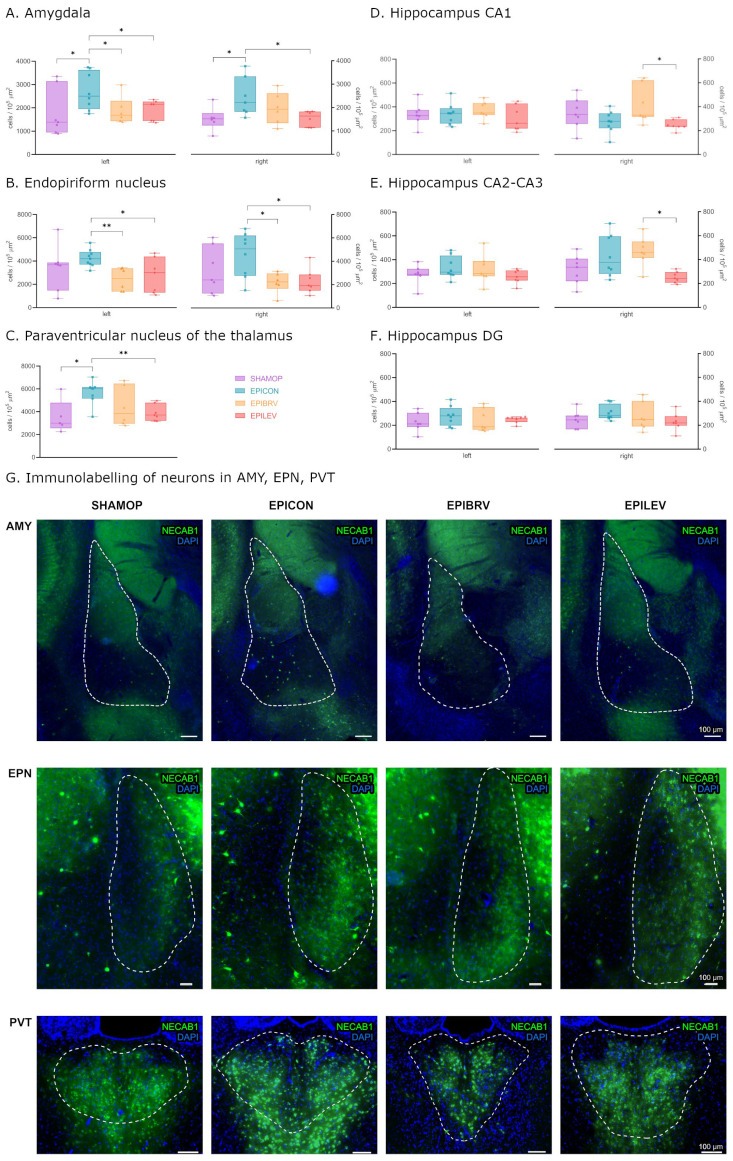
Density of only NECAB1+ cells in several brain regions in epileptic conditions and with ASM treatment. Statistical analysis of cell density in the amygdala (**A**), endopiriform nucleus (**B**), paraventricular nucleus of the thalamus (**C**), hippocampus CA1 (**D**), hippocampus CA2–CA3 (**E**), and hippocampus DG (**F**) brain regions. Fluorescent immunolabelling of neurons PVT, AMY, and EPN brain regions (**G**). Sham-operated group (SHAMOP), epileptic control group (EPICON), brivaracetam-treated group (EPIBRV), and levetiracetam-treated group (EPILEV). An alpha value of 0.05 was used as the cutoff for significance, and we used the following notations: * *p* < 0.05, ** *p* < 0.01.

In order to examine whether the expression levels of NECAB1, PV, and CR are influenced by circuit reorganization caused by temporal lobe epilepsy and how novel antiseizure medication modulates these changes, we first quantified NECAB1-immunopositive neuron cell bodies across distinct brain regions, including the amygdala, endopiriform nucleus, and the paraventricular nucleus of the thalamus and the hippocampus (CA1, CA2–CA3, and dentate gyrus; see Figure 2).

Six weeks after the initial insult (ICV KA injection to the right lateral ventricle followed by status epilepticus), we observed a significant increase in NECAB1-positive cell density in the amygdala on both hemispheres in the epileptic control group compared to sham-operated controls (left—contralateral to KA injection: 2707 ± 284.5 vs. 1781 ± 387.3 cells/10^5^ μm^2^, *p* = 0.04; right—ipsilateral to KA injection: 2442 ± 311.7 vs. 1520 ± 203.1 cells/10^5^ μm^2^, *p* = 0.033) and PVT (5642 ± 415.2 vs. 3536 ± 647.5 cells/10^5^ μm^2^, *p* = 0.045). Treatment with levetiracetam or brivaracetam markedly attenuated this effect, reducing the number of NECAB1-positive cells in both the ipsilateral (right: EPILEV vs. EPICON *p* = 0.028, and EPIBRV vs. EPICON *p* = 0.284) and contralateral (left: EPIBRV vs. EPICON *p* = 0.047, EPILEV vs. EPICON *p* = 0.03) amygdalar nuclei. In contrast to levetiracetam, pharmacological treatment with brivaracetam had only a modest impact on NECAB1-positive cell density changes in the PVT when compared to the epileptic control (EPILEV vs EPICON 3903 ± 270.7 vs. 5642 ± 415.2 cells/10^5^ μm^2^, *p* = 0.004; EPIBRV vs. EPICON *p* = 0.178) (Figure 2A,C,G). Although the increase in cell numbers in the endopiriform nucleus of the EPICON group did not reach statistical significance compared to SHAMOP (left *p* = 0.313, right: *p* = 0.209), both ASM treatment groups significantly lowered cell density when compared to the epileptic control group (left: EPIBRV vs. EPICON *p* = 0.004, EPILEV vs. EPICON *p* = 0.04; right: EPIBRV vs EPICON *p* = 0.012, EPILEV vs. EPICON *p* = 0.016). We did not find significant differences between the SHAMOP and ASM-treated groups (Figure 2B,G).

An analysis of the hippocampal subregions revealed no substantial effect of KA on NECAB1-positive cell numbers. However, we observed a significant difference between the brivaracetam- and levetiracetam-treated groups in the right CA1 (415.3 ± 59.55 vs. 247.2 ± 16.32 cells/10^5^ μm^2^, *p* = 0.049) and CA2–CA3 (471.5 ± 46.93 vs. 252.3 ± 17.89 cells/10^5^ μm^2^, *p* = 0.018) regions but not in the dentate gyrus (*p* = 0.787), corresponding to the side of the KA injection. Notably, there was a significantly higher density of NECAB1-positive cells (unpaired *t*-test with Welch’s correction, *p* = 0.044) in the right CA2–3 region of the hippocampus when compared to the contralateral side (Figure 2D–F).

Next, we investigated changes in PV-positive and NECAB1–PV colocalizing cells caused by KA injection and pharmacological interventions. In the amygdalar nuclei of the EPICON group, both solely PV-positive and NECAB1–PV colocalizing interneurons exhibited a significant hemispheric asymmetry, showing a higher cell density on the left side (for PV-positive cells: left 473.3 ± 52.71 vs. right 281.6 ± 47.14 cells/10^5^ μm^2^, *p* = 0.018; for NECAB1–PV colocalizing cells: left 39.79 ± 6.97 vs. right 9.13 ± 6.92 cells/10^5^ μm^2^, *p* = 0.011).

### 2.3. Comparative Analysis of NECAB1 Expression Patterns in Brain Regions in the Context of Epilepsy and ASM Treatment

**Figure 3:**
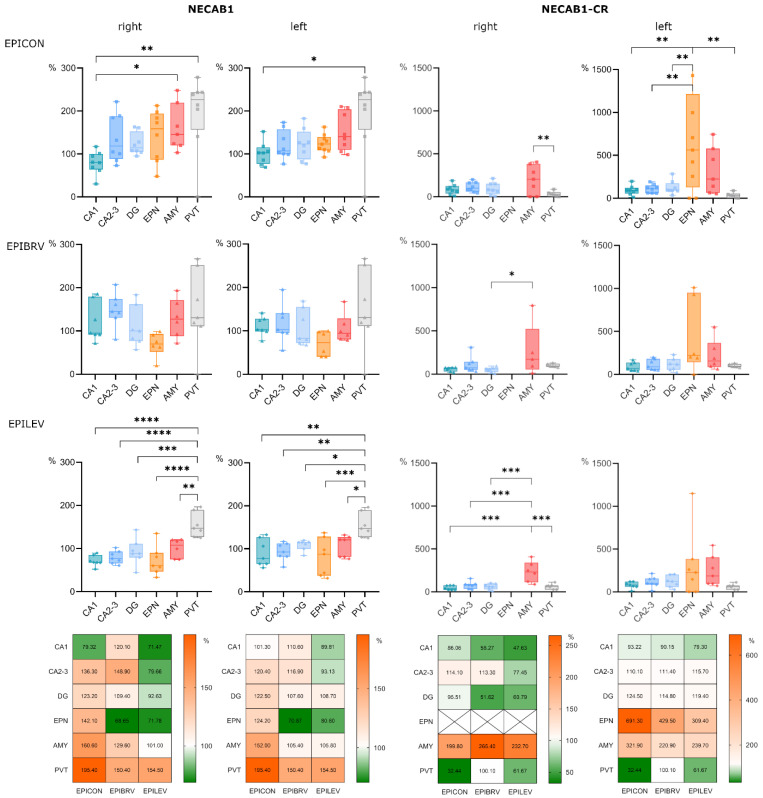
Comparative analysis of NECAB1 expression patterns in brain regions in the context of epilepsy and levetiracetam and brivaracetam treatment. Panels show cell density across different brain regions normalized to the corresponding brain region of the sham-operated group, expressed as a percentage. Bar charts show cell densities separately for the right (ipsilateral to KA injection) and left (contralateral to KA injection) side for the epileptic control (EPICON), brivaracetam-treated epileptic (EPIBRV), and levetiracetam-treated epileptic (EPILEV) groups. The notable exception is the midline nucleus PVT, which is repeated for both sides within an experimental group. Relative changes of cell densities are visualized as heatmaps on the bottom, where 100% (white) corresponds to the cell density of the sham-operated group (for each side, brain region and treatment group). An alpha value of 0.05 was used as the cutoff for significance, and we used the following notations: * *p* < 0.05, ** *p* < 0.01, *** *p* < 0.001, and **** *p* < 0.0001.

Next, we assessed changes in NECAB1+ cell density across different brain regions in epileptic groups, normalized to the corresponding regions in the sham-operated group (Figure 3). The paraventricular nucleus of the thalamus and the amygdala exhibited the most pronounced increases in cell density, with a significant difference when compared to the CA1 region of the hippocampus. These changes were most prominent in the epileptic control (right side: PVT vs. CA1 *p* = 0.002, AMY vs. CA1 *p* = 0.05; left side: PVT vs. CA1 *p* = 0.008) and levetiracetam-treated groups (right side: PVT vs. CA1 *p* < 0.0001; left side: PVT vs. CA1 *p* = 0.002). However, in the levetiracetam-treated group, all brain regions—except the PVT—displayed a lower density of NECAB1-positive cells compared to both the epileptic and brivaracetam-treated groups (right side: F (2, 110) = 9.489, *p* = 0.0002; left side: F (2, 113) = 6.677, *p* = 0.002).

Notably, while the specific subpopulation of NECAB1-positive cells co-expressing calretinin was absent in the right endopiriform nucleus across both treated and untreated epileptic groups, its density was increased in the left endopiriform nucleus compared to all other examined brain regions, with this difference reaching significance in the epileptic control group (F (5, 109) = 9.376, *p* < 0.0001). Furthermore, the density of NECAB1–CR co-expressing cells was significantly higher in the right amygdala compared to other brain areas (F (4, 88) = 10.45, *p* < 0.0001). The same trend could be observed on the left side, albeit this difference did not reach significance (Figure 3).

**Figure 4:**
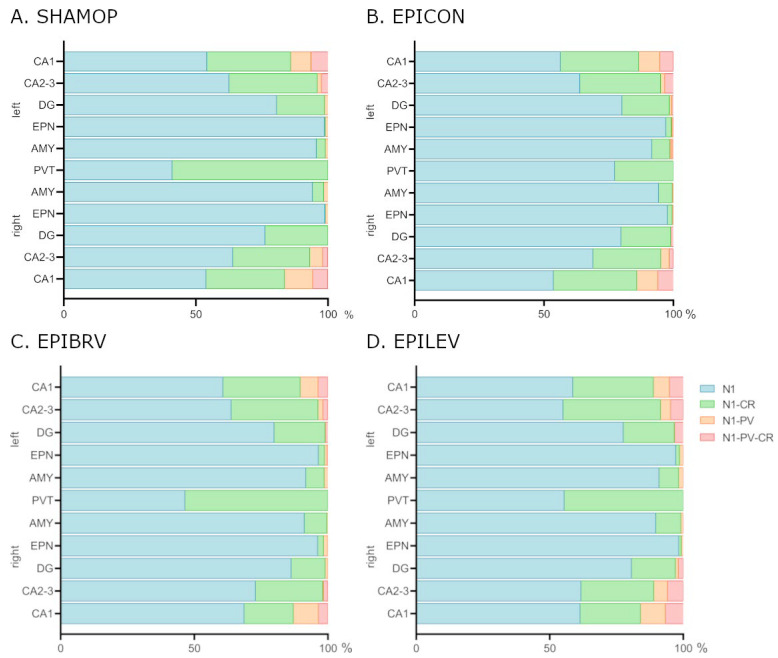
Expression changes of NECAB1 and NECAB1 colocalizing cells in epileptic control and different ASM treatment groups. (**A**) Sham-operated group. (**B**) Epileptic control group. (**C**) Brivaracetam-treated epileptic group. (**D**) Levetiracetam-treated epileptic group. N1 = cells expressing only NECAB1; N1-PV = NECAB1–parvalbumin co-expressing cell; N1-CR = NECAB1–calretinin co-expressing cells; N1-PV-CR = NECAB1–parvalbumin–calretinin co-expressing cells; CA1, CA2, DG = hippocampal areas; EPN = endopiriform nucleus; AMY = amygdala; PVT = paraventricular nucleus of the thalamus.

The ratio of NECAB1 and NECAB1–CR colocalizing cells changed drastically (Figure 4) in the PVT brain region of the epilepsy control group when compared to sham-operated group (Fisher’s exact test, *p* < 0.0001). This expression change was reversed equally by levetiracetam (EPILEV vs. EPICON: *p* = 0.002) and brivaracetam (EPIBRV vs. EPICON: *p* < 0.0001) treatment, with no significant difference between the two ASMs (see Figure 4A–D).

## 3. Discussion

NECAB1 has recently emerged as a calcium-binding protein of interest, serving as a promising neuronal marker. However, its distribution, physiological role, cell type specificity, colocalization with other markers/calcium-binding proteins, and, most importantly, its expression dynamics under pathological conditions remain partially understood.

In this study, we aimed to characterize the expression and distribution of NECAB1, first in the healthy brain, specifically in brain regions known to be involved in the TLE circuitry. For this, we described the normal topological differences in NECAB1 density in the amygdala, endopiriform nucleus, paraventricular nucleus of the thalamus, and in the hippocampal subfields. Afterwards, we examined the distribution changes caused by KA-induced TLE and the possible modulating effects of the anti-epileptic drugs levetiracetam and brivaracetam.

By mapping the expression patterns in the healthy rat brain, we found that cells expressing only NECAB1 were more abundant in the paraventricular nucleus of the thalamus, endopiriform nucleus, and amygdala compared to the hippocampus. Expression patterns observed in this study closely resemble regional expression levels described in transcriptomic data from the Human Protein Atlas and RNA in situ hybridization (ISH) data from the Allen Mouse Brain Atlas for the selected CaBP genes [22].

Recent studies have shown that in the hippocampus, NECAB1 was present in hilar mossy cells as well as interneurons, concentrated at the border between stratum radiatum and stratum lacunosum-moleculare [23]. A similar distribution was observed in the rat hippocampus in our study.

Furthermore, in the hippocampus, basolateral amygdala (BLA), and somatosensory cortex, NECAB1 has been demonstrated to be highly expressed in CB1/CCK-positive GABAergic interneurons, which represent a major subpopulation of inhibitory neurons [12].

In accordance with in silico expression data [12], we described a subpopulation of NECAB1-positive cells co-expressing calretinin, which were present in the amygdala, endopiriform nucleus, and paraventricular nucleus of the thalamus and hippocampus. In comparison to the solely NECAB1-positive cells, these had a significantly lower density in all examined regions.

Additionally, we found a significant difference between the left and the right amygdalar nuclei regarding the NECAB–PV colocalizing neurons and the solely PV-positive cells. This finding aligns with prior reports indicating increased PV-positive cell density in the left amygdala [24]. The presence of colocalizing NECAB1 and PV cells is not surprising, since a subset of neurons in the dLGN (dorsal lateral geniculate nucleus) have been shown to co-express PV and NECAB1, suggesting that some neurons have transitional molecular profiles [25].

NECAB1 expression varies among PV-positive hippocampal cells and is notably absent in parvalbumin-positive axo-axonic cells in the hippocampus, distinguishing them from other parvalbumin-expressing interneurons like basket cells and bistratified cells, where NECAB1 presence varies [26].

To our knowledge, this is the first study to examine NECAB1 expression and colocalization in the endopiriform nucleus. In the healthy brain, we found a significantly higher density of cells expressing only NECAB1 in the EPN compared to hippocampal subregions, a very low density of NECAB1–CR colocalizing cells, and a low density of NECAB1–PV colocalizing cells (around 1% of NECAB1-expressing neurons).

Emerging evidence suggests that NECAB1 expression patterns may delineate subpopulations of cells along a gene expression gradient, as demonstrated in thalamic subnetworks [27]. For example, NECAB1 exhibits enriched expression in specific thalamic subdivisions, including the mediodorsal nucleus and peri-ATN neurons, where it colocalizes with markers like Tnnt1 and Calb2, suggesting a role in thalamic subnetwork organization [27,28,29]. In this study we demonstrate for the first time the presence of NECAB1+ and NECAB1–CR co-expressing cells in the PVT, with a slight predominance of NECAB1–CR cells over only NECAB1-expressing ones in physiological conditions.

Given the complexity of the underlying mechanisms of temporal lobe epilepsy, the identification of even a single factor that advances our understanding of epileptogenesis can be of critical importance. Dysregulation of calcium signaling plays a key role in epileptogenesis, as excessive calcium influx disrupts synaptic transmission and promotes hyperexcitability [30,31].

Calcium-binding proteins serve a crucial buffering function, regulating intracellular calcium levels and maintaining calcium homeostasis [32,33]. The distribution of EF-hand CaBPs in the central nervous system (CNS) is highly heterogeneous; while some are ubiquitously expressed, others exhibit region-specific, cell-type-specific, and/or developmental stage-specific expression patterns [34].

Data about the expression pattern of several well-known calcium-binding proteins in epileptic context are contradictory. Some suggest elevated levels of PV and CR in epileptic tissue in order to potentially counteract seizure activity [35], while others suggest preserved PV+ cell density [36] or interneuron dysfunction and the reduction of PV-positive interneurons, leading to seizure susceptibility [37].

In our study, in the chronic phase of KA-induced epilepsy, we observed an increase in NECAB1 expression in several brain regions, namely the endopiriform nucleus, the amygdala, and the paraventricular nucleus of the thalamus. As NECAB1 is a prominent marker of CCK/CB1 inhibitory interneurons, the increase may be associated with the upregulation of CB1 receptor immunostaining in the hippocampus, a phenomenon documented in both epileptic patients and animal models with CA1 sclerosis [38]. This upregulation of CB1 receptors in the chronic phase of epilepsy is thought to represent a neuroprotective mechanism aimed at reducing neuronal excitability and hyper-synchronization by modulating the release of glutamate and GABA [39]. Furthermore, acute and chronic seizures have been shown to elevate CB1-receptor immunostaining in key limbic structures, including the inner molecular layer of the hippocampus and amygdala, as demonstrated in genetic models of epilepsy [40]. Interestingly, while the activation of muscarinic acetylcholine receptors (mAChRs) typically inhibits GABA release from CB1-expressing terminals, mAChR activation can also excite CCK/CB1 co-expressing interneurons, adding complexity to the regulatory mechanisms involved in epilepsy [41]. However, it must be noted that in the human hippocampus of patients with chronic, intractable TLE, CB1 was shown to be downregulated in glutamatergic boutons [42].

It is noteworthy to mention the striking difference in our findings regarding NECAB1–CR co-expressing cells between the right and left EPN in epileptic groups: these cells were missing completely from the right endopiriform nucleus (ipsilateral to the KA injection) whilst having a high density on the left side (significantly higher in the epileptic control group when compared to all other examined brain regions).

Calretinin-expressing interneurons have previously been shown to be highly vulnerable to excitotoxicity in the epileptic brain, particularly in regions such as the hippocampus, amygdala, neocortex, and thalamus. This susceptibility contributes to the disruption of inhibitory circuits, which plays a critical role in the pathophysiology of epilepsy. In the amygdala, the activation of CR neurons can lead to the disinhibition of pyramidal neurons in the lateral nucleus, potentially facilitating the synchronization of neuronal activity and promoting seizure generation [43]. Furthermore, studies have demonstrated a significant loss and reorganization of CR-containing interneurons in epileptic tissue, with the extent of cell loss correlating with the severity of principal neuron degeneration [44]. Additionally, CR-immunoreactive neurons, which are integral components of the inhibitory system in regions such as the piriform cortex and endopiriform nucleus, are particularly susceptible to excitotoxic damage, further exacerbating the imbalance between excitation and inhibition in epilepsy [45]. Our findings suggest that NECAB1–calretinin cells are similarly affected in epilepsy.

Certain antiseizure medications primarily exert their effects by targeting voltage-dependent calcium channels (VDCCs) rather than directly modulating calcium-binding proteins. Nevertheless, their influence on calcium signaling pathways can indirectly alter CaBP activity by regulating calcium influx [31]. Currently, there are limited data available on the impact of ASMs that do not target calcium channels on the expression or functional dynamics of calcium-binding proteins.

The novel ASMs levetiracetam and brivaracetam, which target the synaptic vesicle glycoprotein 2A (SV2A) [46,47], have been shown to participate in the reorganization of the epileptic tissue [48], but they have not yet been thoroughly investigated regarding their effects on interneurons and calcium-binding proteins.

The direct or indirect link between NECAB1 and SV2A in calcium-dependent processes remains to be determined. However, both interact with synaptotagmin-1 (Syt1), the primary calcium sensor for vesicle fusion, which is essential for fast, synchronous neurotransmitter release [23].

SV2A is a synaptic vesicle transmembrane protein critical for regulating neurotransmitter release and synaptic vesicle recycling. By interacting with synaptotagmin-1, it ensures that Syt1 is properly packaged into synaptic vesicle membranes during endocytosis. Disruption of SV2A leads to Syt1 mislocalization and impaired neurotransmission, a mechanism that may contribute to epilepsy [49].

To date, NECAB1 has been identified as the only binding protein for the C2-domain of synaptotagmin-1 in vitro, suggesting a specific interaction that could modulate the role of Syt1 in calcium-dependent neurotransmitter release. Even though NECAB1 does not share the presynaptic localization of synaptotagmin [12], it could be assumed that NECAB1 might transiently associate with synaptotagmin during vesicle recycling or calcium signaling.

In this study, we demonstrated that while epilepsy increased the density of NECAB1-positive cells, animals treated with either levetiracetam or brivaracetam exhibited cell densities comparable to those observed in the sham-operated control group. These results suggest that although circuit reorganization occurs within the first six weeks following epilepsy induction in rats, such changes are less pronounced in animals receiving ASM treatment. Notably, NECAB1–calretinin co-expressing cells displayed heightened vulnerability across all three epileptic groups, with the most marked reductions observed in the paraventricular thalamic nucleus and the right endopiriform nucleus. The examined ASMs did not exert a significant effect on cells expressing the classical calcium-binding proteins parvalbumin and calretinin. These findings highlight the differential impact of SV2A-targeting ASMs on interneuronal subpopulations with specific calcium-binding protein expression patterns in epileptic conditions.

In contrast to parvalbumin-positive and calretinin-positive interneurons, which in prior studies have shown contradictory results to either remain stable, increase, or decrease in epilepsy six weeks after kainic acid-induced status epilepticus, we found a significant increase in NECAB1-positive cell density in the amygdala bilaterally and the paraventricular nucleus of thalamus of the epileptic control group compared to sham-operated animals. Both levetiracetam and brivaracetam treatment markedly attenuated this increase, significantly reducing NECAB1-positive cell density in the amygdala and endopiriform nucleus. Notably, while NECAB1+ cell density also increased in the endopiriform nuclei in both hemispheres, statistical significance was only observed when comparing the epileptic control group to the ASM-treated groups.

Interestingly, in the PVT, the ratio between NECAB1+ and NECAB1-colocalizing cells significantly shifted in the epileptic group towards the predominance of solely NECAB1-expressing cells compared to the NECAB1–calretinin co-expressing ones. These changes could not be observed in the case of the ASM-treated groups. According to Schurmans et al., calretinin expression in interneurons maintains low intracellular calcium concentrations, preventing calcium overload that can lead to neuronal damage, and helps regulate the excitability of dentate granule cells [50]. Our results suggest that during epilepsy there may be a downregulation of these critical proteins, which may be reversed by certain ASMs.

In pathological conditions, neurons co-expressing multiple calcium-binding proteins can dynamically regulate the expression of these different CaBPs, with evidence supporting both upregulation and downregulation of specific proteins depending on the context. For example, in surviving neurons during TLE, calbindin-D28k is often downregulated, while parvalbumin may be upregulated, possibly as a compensatory mechanism to counteract excessive calcium influx [51,52]. Hippocampal sclerosis shows loss of calretinin-positive interneurons but preservation (or upregulation) of PV-expressing cells, suggesting selective vulnerability [53].

Given that different cells can be found along a gene expression gradient, the increase of NECAB1 in the co-expressing cells may show capacity for CaBP expression shifts under changing physiological cellular demands or during pathological adaptations.

Although the brain regions explored in this study represent key components of the epileptic circuitry, examination of changes in NECAB1-positive neurons in other areas warrants further investigation. NECAB1 has been demonstrated to show strong immunoreactivity and participate in synchronized network activity in the entorhinal cortex [54], and NECAB1-positive cells, either alone or in combination with other markers like cholecystokinin (CCK) or special AT-rich sequence-binding protein-1 (SATB1), are among the diverse types of GABAergic interneurons innervated by septal projections [55]. However, their alterations under epileptic conditions are yet to be determined.

## 4. Materials and Methods

### 4.1. Ethical Approval and Compliance

The experiments were performed in accordance with the ethical code of the 2010/63/EU Directive of the European Parliament and national regulations, and they were approved by the Ethics Committee for Scientific Research of the George Emil Palade University of Medicine, Pharmacy, Science, and Technology of Târgu Mureș (UMFSTGEP; license no. 43/06.03.2020 and 52/31.03.2022).

### 4.2. Animals

Experimentally naïve, young adult (8-week-old) male Wistar rats were obtained from the Laboratory Animal Core Facility of UMFSTGEP. The animals underwent a 5-day acclimatization period to single-housing conditions and daily handling. They were maintained under standard environmental conditions, including a 12-h light–dark cycle, a room temperature of 20 ± 2 °C, and a humidity level of 60% ± 10%. The rats were housed in standard polypropylene cages (1291H Eurostandard Type III H, 425 mm × 266 mm × 185 mm, Techniplast, Milan, Italy) and had ad libitum access to standard rodent pellet chow (“Cantacuzino” National Institute of Research and Development, Bucharest, Romania) and tap water. At the end of the acclimatization period, the animals were randomly distributed into sham-operated (SHAMOP, n = 7), epileptic control (EPICON, n = 8), epileptic brivaracetam-treated (EPIBRV, n = 7), and epileptic levetiracetam-treated (EPILEV, n = 7) groups.

### 4.3. Epilepsy Induction

The intracerebroventricular kainic acid model was utilized to induce chronic temporal lobe epilepsy [56]. For this, animals were deeply anesthetized with 5% isoflurane (Anesteran, Rompharm Company SRL, Otopeni, Romania) using the EZ-SA800 Single Animal Anesthesia System (World Precision Instruments Inc., Sarasota, FL, USA), with a 0.5–2.0% maintenance dose, based on monitorization of anesthesia depth. The animals were positioned in a stereotaxic frame (Digital Stereotaxic with Manual Fine Drive, Leica Biosystems, Buffalo Grove, IL, USA) on a heating pad (Physiological Temperature Controller TMP5b, Supertech, Pécs, Hungary). The syringe was positioned according to the Rat Brain Atlas by Paxinos and Watson [57]. Epileptic experimental groups received 0.6 μg of KA (Sigma Aldrich, St. Louis, MO, USA) dissolved in 2 μL of 0.9% saline solution. The sham-operated group received 2 μL 0.9% saline solution. Injections were administered into the right lateral ventricle (coordinates = AP: −0.7 mm, ML: +1.5 mm, and DV: 3.6 mm from skull surface) using a 10 μL Hamilton syringe (Hamilton Company, Reno, NV, USA) connected to a motorized programmable stereotaxic injector (Stoelting QSI Model 5311, Stoelting Co., Wood Dale, IL, USA). Injection speed was set to 0.4 µL/min, and the needle was left in place for 10 min to ensure proper diffusion. Upon recovery from anesthesia, KA-treated animals exhibited tonic-clonic seizures, which escalated to status epilepticus, with an approx. 30% mortality rate. Seizure activity was assessed using the revised Racine’s scale, and only those animals that displayed seizures of Racine stage 5–6 severity within three hours of induction were included in the study [58]. After recovering and receiving intraperitoneal rehydration, the animals were returned to their home cages.

### 4.4. Pharmacological Interventions

Three weeks after the induction with KA, EPIBRV and EPILEV groups received oral anti-epileptic medication for 21 days via an automated drug-pellet distribution system installed in their home cages, as developed by Bába et al. [59]. Drug administration was preceded by a 5-day habituation and training period, during which all animals were conditioned to consume pellets from the distribution system. Following habituation, drug-infused pellets were provided in 12-h intervals, delivering doses of 60 mg/kg body weight for brivaracetam and 70 mg/kg body weight for levetiracetam. To prepare the pellets, the drugs were blended with lactose and glucose powder (1 g each), and glucose syrup was added for consistency. Pellet consumption was monitored daily, to ensure constant and complete pellet intake throughout the experiment.

### 4.5. Tissue Processing for Immunohistochemistry

At the end of the three-week-long pharmacological treatment period, animals from all treatment groups were deeply anesthetized via intraperitoneal injection of a ketamine (100 mg/kg) and xylazine (10 mg/kg) mixture, followed by transcardial perfusion with ice-cold 0.9% saline. For brain tissue fixation, a solution containing 4% paraformaldehyde (PFA; Sigma Aldrich, St. Louis, MO, USA) and 15% picric acid (Sigma Aldrich, St. Louis, MO, USA) in 0.1 M phosphate buffer (PB, pH 7.4; Sigma Aldrich, St. Louis, MO, USA) was perfused for 20 min. Brains were collected and post-fixed in 4% PFA for 24 h.

### 4.6. Immunohistochemical Staining

For immunohistochemical staining, serial coronal sections (60 μm-thick) were cut in 0.1 M PB using a vibratome (VT 1000S, Leica, Nussloch, Germany). Triple fluorescent immunostaining was performed to label NECAB1-positive, parvalbumin-positive and calretinin-positive cells. The staining protocol began with a 10-min wash of free-floating dorsal hippocampal slices in 0.1 PB, followed by two additional 10-min washes in tris-buffered saline (TBS; Sigma-Aldrich, St. Louis, MO, USA). To block nonspecific binding sites, sections were incubated for 45 min in a solution of 10% normal horse serum (NHS; Vector Laboratories, Burlingame, CA, USA) and 0.3% Triton-X (Sigma-Aldrich, St. Louis, MO, USA) in TBS. Overnight incubation at room temperature was carried out with primary antibodies against NECAB1 (anti-NECAB1 = rabbit—polyclonal, dilution 1:500, production no: HPA023629, Atlas Antibodies, Stockholm, Sweden), PV (anti-PV = goat—dilution 1:500, production no: PVG-214, Swant, Barron, WI, USA) and CR (anti-CR = mouse—monoclonal, dilution 1:500, production no: MAB1568, Millipore, Burlington, MA, USA) dissolved in TBS containing 0.1% Triton-X. On the second day, sections underwent a single TBS wash cycle before incubation with secondary antibodies (Alexa 488 = conjugated donkey anti-rabbit, dilution 1:400, 711-545-152, Jackson ImmunoResearch Laboratories, West Grove, PA, USA; Alexa 647 = conjugated donkey anti-goat, dilution 1:400, 705-605-147 Jackson ImmunoResearch Laboratories, West Grove, PA, USA; Alexa 594 = conjugated donkey anti-mouse, dilution 1:400, 715-585-150, Jackson ImmunoResearch Laboratories, West Grove, PA, USA) and DAPI (Sigma, 5 087 410 001) for 4 h at room temperature. This was followed by sequential washes in TBS and PB before mounting. Slides were coverslipped with Vectashield mounting medium (Vector Laboratories, Burlingame, CA, USA) and sealed with nail polish.

### 4.7. Image Acquisition and Analysis

Images of immunostained slices were acquired using a 3DHISTECH Pannoramic MIDI II slide scanner provided by the HUN-REN Institute of Experimental Medicine, Budapest, Hungary. A Zeiss Plan-Apochromat 20X dry objective was used, along with Cy3.5, FITC-2024B, LF 635-C, and DAPI-Q filters. For image analysis, regions of interest (ROI) were manually delineated in ImageJ software (version 1.54g, https://imagej.net/ij/) using the Paxinos and Watson Rat Brain Atlas as references. Areas with tissue tears, autofluorescence, or non-specific signals were excluded during image preprocessing and background subtraction. Manual cell quantification was performed in the CA1, CA2, CA3, and DG hippocampal subregions, paraventricular nucleus of the thalamus (PVT), amygdala (AMY), and endopiriform nucleus (EPN), with cell density expressed as the number of cells per 10^5^ μm^2^.

### 4.8. Statistical Analysis

Image and statistical analyses were performed by an experimenter blinded towards the treatment groups. GraphPad Prism 8 software (version 8.0.1, GraphPad Software Inc., San Diego, CA, USA) was used for statistical analysis. All data were expressed as the mean ± standard error of the mean (S.E.M). Prior to statistical comparisons, all data were checked using the robust regression and outlier removal method for multiple outliers’ detection. The Kolmogorov–Smirnov test was used to test the data for normality and the appropriate statistical method. For matched observations, a two-way ANOVA for unmatched groups was used, and in case of data with a Gaussian distribution, a one-way ANOVA was used; in both cases, this was followed by Tukey’s post hoc multiple comparison test. In case of non-Gaussian distribution, the Kruskal–Wallis nonparametric test with Dunn’s multiple comparisons test was used. An alpha value of 0.05 was used as the cutoff for significance, and we used the following notations: * *p* < 0.05, ** *p* < 0.01, *** *p* < 0.001, and **** *p* < 0.0001.

## 5. Conclusions

Calcium-binding proteins are essential for calcium homeostasis and may serve as therapeutic targets.

In our study, in healthy brain areas otherwise involved in the epileptic circuitry, NECAB1-positive cells were most abundant in the paraventricular nucleus of the thalamus, endopiriform nucleus, and amygdala. A subpopulation of cells co-expressing calretinin was found in the amygdala, PVT, and hippocampus but was nearly absent in the endopiriform nucleus. In the chronic phase of KA-induced epilepsy, NECAB1 expression was significantly upregulated in the PVT and bilaterally in the amygdala. In epileptic rats, NECAB1–CR co-expressing cells were absent in the right endopiriform nucleus but had a high density on the left, whereas the NECAB1–PV colocalizing cells showed an asymmetry between the right and left amygdala.

These findings suggest that NECAB1 upregulation may be a compensatory response to epileptic hyperexcitability. Given its involvement in thalamocortical regulation and interneuron diversity, NECAB1 may play a critical role in circuit remodeling. We also found that levetiracetam and brivaracetam treatments had a substantial modulating effect on NECAB1 expression changes, leading to partial reduction of density increase caused by TLE.

Further studies are needed to elucidate the role of NECAB1-positive neurons in epileptic seizure susceptibility and disease progression. Understanding NECAB1’s role in epilepsy and neurodegeneration could open new therapeutic avenues targeting calcium-binding proteins for neuronal stability and excitability regulation.

## Figures and Tables

**Table 1:** NECAB1 and colocalizing cells’ proportional distribution in the investigated brain regions of sham-operated (SHAMOP) animals. Left: contralateral to the KA injection; right: ipsilateral to the KA injection site. N1 = cells expressing only NECAB1; N1-PV = NECAB1–parvalbumin co-expressing cells; N1-CR = NECAB1–calretinin co-expressing cells; N1-PV-CR = NECAB1–parvalbumin calretinin co-expressing cells; CA1, CA2, and DG = hippocampal areas; EPN = endopiriform nucleus; AMY = amygdala; PVT = paraventricular nucleus of the thalamus.

%	Left						Right				
	CA1	CA2-3	DG	EPN	AMY	PVT	AMY	EPN	DG	CA2-3	CA1
**N1**	54.25	62.59	80.62	98.69	95.64	41.00	94.13	98.91	76.23	64.03	53.84
**N1-PV**	7.67	1.53	1.28	0.92	0.94	0.00	1.58	1.09	0.00	4.87	10.64
**N1-CR**	31.69	33.44	18.10	0.39	3.42	59.00	4.29	0.00	23.77	29.11	29.78
**N1-PV-CR**	6.40	2.44	0.00	0.00	0.00	0.00	0.00	0.00	0.00	1.98	5.74

## Data Availability

The data presented in this study are available on request from the corresponding author.

## Abbreviations

The following abbreviations are used in this manuscript:

AMY	Amygdala
ASMs	Antiseizure medication
BRV	Brivaracetam
CaBPs	Calcium-binding proteins
CB1	Cannabinoid receptor 1
CCK	Cholecystokinin
CNS	Central nervous system
CR	Calretinin
DG	Dentate gyrus
EPIBRV	Brivaracetam-treated
EPICON	Epileptic control
EPILEV	Levetiracetam-treated
EPN	Endopiriform nucleus
GABA	Gamma-aminobutyric acid
KA	Kainic acid
LEV	Levetiracetam
mAChRs	Muscarinic acetylcholine receptors
N1	NECAB1
N1-CR	NECAB1–calretinin co-expressing cells
N1-PV	NECAB1–parvalbumin co-expressing cell
N1-PV-CR	NECAB1–parvalbumin–calretinin co-expressing cells
NECAB1	N-Terminal EF-Hand Calcium-Binding Protein 1
PV	Parvalbumin
PVT	Paraventricular nucleus of the thalamus
SHAMOP	Sham-operated group
SV2A	Synaptic vesicle glycoprotein 2A
Syt1	Synaptotagmin-1
TLE	Temporal lobe epilepsy
